# Mangroves Enhance Reef Fish Abundance at the Caribbean Regional Scale

**DOI:** 10.1371/journal.pone.0142022

**Published:** 2015-11-04

**Authors:** Joseph E. Serafy, Geoffrey S. Shideler, Rafael J. Araújo, Ivan Nagelkerken

**Affiliations:** 1 Southeast Fisheries Science Center, National Marine Fisheries Service, Miami, Florida, United States of America; 2 Rosenstiel School of Marine & Atmospheric Science, University of Miami, Miami, Florida, United States of America; 3 Southern Seas Ecology Laboratories, School of Biological Sciences and The Environment Institute, University of Adelaide, Adelaide, Australia; Texas A&M University at Galveston, UNITED STATES

## Abstract

Several studies conducted at the scale of islands, or small sections of continental coastlines, have suggested that mangrove habitats serve to enhance fish abundances on coral reefs, mainly by providing nursery grounds for several ontogenetically-migrating species. However, evidence of such enhancement at a regional scale has not been reported, and recently, some researchers have questioned the mangrove-reef subsidy effect. In the present study, using two different regression approaches, we pursued two questions related to mangrove-reef connectivity at the Caribbean regional scale: (1) Are reef fish abundances limited by mangrove forest area?; and (2) Are mean reef fish abundances proportional to mangrove forest area after taking human population density and latitude into account? Specifically, we tested for Caribbean-wide mangrove forest area effects on the abundances of 12 reef fishes that have been previously characterized as “mangrove-dependent”. Analyzed were data from an ongoing, long-term (20-year) citizen-scientist fish monitoring program; coastal human population censuses; and several wetland forest information sources. Quantile regression results supported the notion that mangrove forest area limits the abundance of eight of the 12 fishes examined. Linear mixed-effects regression results, which considered potential human (fishing and habitat degradation) and latitudinal influences, suggested that average reef fish densities of at least six of the 12 focal fishes were directly proportional to mangrove forest area. Recent work questioning the mangrove-reef fish subsidy effect likely reflects a failure to: (1) focus analyses on species that use mangroves as nurseries, (2) consider more than the mean fish abundance response to mangrove forest extent; and/or (3) quantitatively account for potentially confounding human impacts, such as fishing pressure and habitat degradation. Our study is the first to demonstrate at a large regional scale (i.e., the Wider Caribbean) that greater mangrove forest size generally functions to increase the densities on neighboring reefs of those fishes that use these shallow, vegetated habitats as nurseries.

## Introduction

Over 25 years ago, Parrish [1] was among the first to highlight the need to quantify interactions between shallow water habitats and offshore systems, including the recruitment of young fishes from mangrove forests and seagrass beds to adult populations on coral reefs. Several recent studies seeking insight into the nursery role of mangroves on Caribbean reef fish abundance indicate a sometimes dramatic enhancement effect on reef fish quantities, depending on the fish taxa, reefs and mangrove stands examined [2–6]. However, a common characteristic of these studies is their limited spatiotemporal scope; each was conducted within or among a small set of islands or continental coastlines, and mostly over periods of fewer than two years. Also, while all prior studies share the use of some form of visual fish survey to gather data, factors such as site choice, analysis, and other methodological differences suggest there is currently a less-than-solid basis for extending previous study findings to different locations and/or generalizing at the regional scale [7].

Among the many factors that differ according to study site is the magnitude of human influence from one location to the next. While Halpern [4] and Mumby et al. [5] each acknowledged it as a possible factor, neither they, nor any of the other studies, directly accounted for (i.e., incorporated in their data analyses) the potentially confounding influence of human activity (i.e., fishing and/or habitat degradation) when examining for relationships linking mangrove presence or quantity to fish abundance on reefs. Although he did not examine a mangrove effect, Stallings [8] found human influence on large, high–trophic level Caribbean fishes to be strong, and a recent global index of ocean health [9] was found to be negatively correlated with coastal human population size.

**Fig 1:**
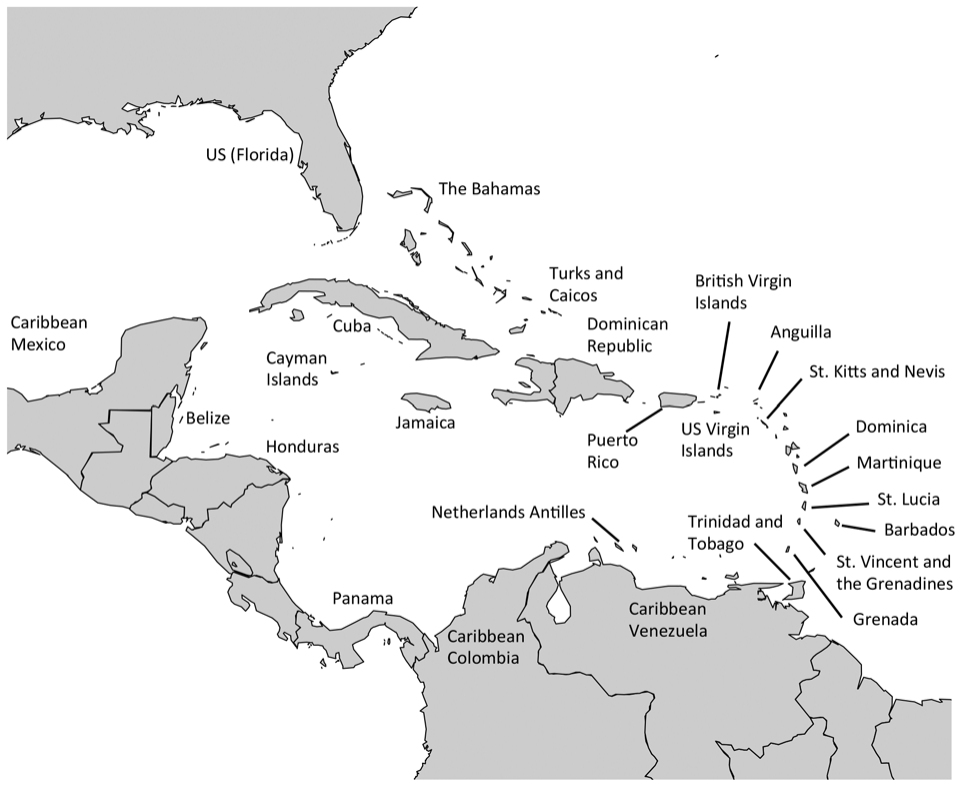
Map of the Wider Caribbean Region with 25 countries/island nations that were included in the final analyses investigating the effect of mangrove forest area on reef fish abundance.

In this paper, we analyzed data from an extensive, fishery-independent, citizen-based fish survey that spans reefs throughout the Wider Caribbean Region (WCR) from Florida, USA, south to Venezuela (Fig 1). Our goal was to examine variation in the abundances on reefs of 12 fishes that were identified as either “highly” or “possibly” mangrove-dependent by Nagelkerken et al. [2]. Our study addressed two important questions. The first was to revisit the question posed by Halpern [4]: Are mangroves a limiting resource for coral reef fishes? Rather than using Halpern’s [4] conventional correlation analysis approach, we addressed his query using quantile regression—a method that is increasingly being used to address questions of resource limitation in ecology [10–14]. Briefly, quantile regression allows analysts to assess more than just the central tendency response of a dependent variable, such as fish abundance, along independent variable gradients, such as measures of habitat quantity or quality [12].

Our second objective was to examine the degree to which the variables, mangrove forest area, human population density, and latitude, were predictive, whether singly or in combination, of the mean abundances of the 12 focal fishes. In this case, we used a linear-mixed effects regression approach. This technique differs from the quantile regression approach in that mixed-effects regression fits to the mean response, whereas quantile regression is conducive to revealing relationships at multiple portions of the response distribution, such as its lower and upper edges (e.g., 10^th^ and 90^th^ quantiles). The present study is unlike previous investigations of the role of mangroves on reef fish abundance in that it: (1) spans the entire Caribbean region; (2) examines for effects across a broad mangrove extent gradient; (3) uses an analytical approach well-suited to address the question of whether mangroves are resources that can limit coral reef fish abundances; and (4) explicitly incorporates mangrove forest area, human population density and latitude into analyses.

## Methods

### Indices of Fish Abundance

Our target species were 12 reef fishes with juvenile life phases that partly, or predominantly, occur in mangrove habitats as identified by Nagelkerken et al. [2]. We here refer to these species as “mangrove-dependent reef fishes.” These included several species within each of the families Lutjanidae, Scaridae, and Haemulidae, in addition to single species belonging to the families Sphyraenidae and Gerreidae (Table 1). To investigate Caribbean-wide patterns of mangrove-dependent reef fish abundance, we utilized the citizen science-based Reef Environmental Education Foundation (REEF) data set. The REEF program utilizes a “roving-diver” technique [15, 16], whereby trained divers volunteer to survey the reefs throughout the Caribbean region and record fish abundances using the scale: 0 = absent, 1 = 1, 2 = 2–10, 3 = 11–100, 4 = more than 100 individuals. No fish size information is collected. Since the REEF’s 1993 inception through 2012, approximately 49,500 fish surveys (after subsetting the data to divers REEF certified and categorized as “expert”), performed by trained volunteer divers using the same roving diver technique, have been logged throughout the WCR. For our analysis, REEF data were summarized for each country or island nation (Fig 1) following the methodology of Wolf and Pattengill-Semmens [17]. Specifically, each species’ sighting frequency (a proportion) per year-country was multiplied by its mean abundance when present, thereby yielding an annual index of fish abundance for each focal species for each country and year. No fish abundance index values were computed for years in which fewer than 10 roving diver surveys per country were performed (see S1 Table for summary of dive survey effort).

**Table 1:** Quantile (90^th^) regression results (model coefficients with standard errors in parentheses) describing the best approximating (based on Akaike information criteria, see text for details) linear relationships between mean index of reef fish abundance and latitude; the natural logarithm of mangrove extent (hectares); or the natural logarithm of human population density (people km^–2^). na = variable not best approximating model.

Family/Species/Common name	Intercept	Latitude	Mangrove extent	Human population density
**Lutjanidae**				
*Lutjanus griseus* (gray snapper)	−0.97 (0.36)	na	0.29 (0.03)	na
*Lutjanus apodus* (schoolmaster)	1.34 (0.20)	na	0.10 (0.02)	na
*Lutjanus analis* (mutton snapper)	−1.21 (0.38)	na	0.12 (0.04)	na
*Ocyurus chrysurus* (yellowtail snapper)	4.41 (0.42)	na	na	−0.28 (0.10)
**Gerreidae**				
*Gerres cinereus* (yellowfin mojarra)	−2.01 (0.52)	na	na	0.65 (0.11)
**Haemulidae**				
*Haemulon parra* (sailors choice)	−1.79 (0.41)	na	0.26 (0.04)	na
*Haemulon sciurus* (bluestriped grunt)	−0.81 (0.25)	na	0.32 (0.02)	na
*Haemulon plumierii* (white grunt)	−1.13 (0.39)	na	0.31 (0.03)	na
**Scaridae**				
*Scarus iseri* (striped parrotfish)	4.00 (0.37)	−0.04 (0.02)	na	na
*Scarus guacamaia* (rainbow parrotfish)	−1.55 (0.43)	na	0.10 (0.05)	na
*Scarus coeruleus* (blue parrotfish)	−2.38 (0.33)	na	0.17 (0.03)	na
**Sphyraenidae**				
*Sphyraena barracuda* (great barracuda)	−0.95 (0.23)	0.07 (0.01)	na	na

### Mangrove Cover Estimates

For each country or island nation in the WCR, an annual mangrove forest area estimate was obtained using values published in the United Nations Food and Agriculture Organization (FAO) Forestry Department Country Reports [18, 19]. The reports constitute a global attempt to quantify total mangrove cover by geographical region. For each country, FAO has identified the most reliable mangrove estimates from various field and remote sensing surveys and these estimates are the basis of country-specific formulas that FAO has calculated to quantify the trajectory and magnitude of mangrove area change between surveys [18, 19]. Thus, we used these FAO formulas to generate annual, country-specific mangrove forest area estimates for each year that REEF fish data was available.

### Human Population Density Estimates

Human population density was used as a proxy for anthropogenic influence on each country’s coastal system and these were estimated for each country-year combination. For most of the countries and island nations, population estimates were available directly via the World Bank database (http://data.worldbank.org/indicator/SP.POP.TOTL) or the CIA World Factbook database (https://www.cia.gov/library/publications/the-world-factbook/). For three countries (Mexico, Colombia, Venezuela), which are relatively large and have significant areas not associated with the Caribbean region (e.g., bordering the Pacific Ocean or far inland), coastal human population estimates were obtained by identifying the nation’s Caribbean and/or Gulf of Mexico coastal states and obtaining human population estimates from appropriate government sources [Instituto Nacional de Estadística y Geografía (Mexico), Departamento Administrativo Nacional de Estadística (Colombia), Instituto Nacional de Estadística (Venezuela)]. To calculate human population density, the population estimate for each year was divided by each country’s size (km^2^, obtained via the CIA World Factbook), with the exception of Mexico, Colombia and Venezuela, which entailed dividing by the areas of their coastal states. In the case of the United States of America, all information was restricted to the State of Florida (to match the REEF data set and mangrove areal extent information).

### Regression Modeling

Data analysis was performed using two different techniques to investigate possible relationships between mangrove forest area and reef fish abundance. First, we used quantile regression modeling (QRM) to examine for evidence that reef fish abundances were *limited* by mangrove forest area, as compared to human population density and latitude. Second, we used linear mixed-effects modeling (LMM) to assess whether a mangrove forest area effect on *mean* fish abundances emerged after accounting for coastal human population density and latitude. In both cases, we sought to reveal (select) those models that best approximated the observed patterns of species-specific abundance. Following Burnham and Anderson [20] selection of best approximating models was based on Akaike information criterion (AIC) raw values and their weights. AIC weights indicate the probability that a particular model is the best model within the suite of models evaluated. Delta AIC value differences <2 were considered to lend substantial support for a model [20]. All QRM and LMM modeling was conducted using the R packages “quantreg” [21] and “nlme” [22], respectively.

### Quantile Regression Modeling

Quantile regressions provide estimates for linear models fit to any part of a response distribution, including near the upper bounds, and require minimal assumptions about the form of the error distributions along a given independent variable (in our case, habitat) gradient. We performed QRM with species-specific fish abundance as the dependent variable and mangrove forest area, human population or latitude as the independent, based on years for which matching data were available. Prior to analyses, mangrove forest area and human population data were log_e_-transformed and fish abundance data were log_e_(*x*+0.1)-transformed. Latitude values were not transformed. For each species, our focus was on the magnitude and sign of the 90^th^ regression quantile coefficients associated with each of the three models, which had either mangrove forest area, human population density, or latitude as the independent variable.

### Linear Mixed-Effects Modeling

Whereas the QRMs examined the potential association between mangrove forest area and reef fish abundance limitation, LMMs addressed whether mean fish densities were proportional to mangrove forest area after considering coastal human population density and latitude. LMM was chosen because the REEF, mangrove, and human population data constituted repeated observations (measures) for each country in a longitudinal data set with missing data points (years with too few, or nil surveys), thus accounting for the non-independence of observations [23, 24]. In this case, indices of fish abundance, mangrove forest area, and human population density were all rank-transformed [25] as no other transformations resolved problems of non-normality and heteroscedasticity. In these linear mixed models, mangrove forest area, coastal human population density, and latitude were fixed effects and country/state was included as a random effect. Following Burnham and Anderson [20], we began with a set of candidate models that incorporated potential predictors of relative fish abundance (S2 Table). All possible combinations of the three variables (mangrove forest area, population density, and latitude), including models with interaction terms, were examined for a total of 10 candidate models for each species. In addition to computing AIC values, AIC weights and Delta AIC value differences, and adjusted-*R* ^2^ values were calculated for each final model. Although human population densities tended to be lower when mangrove forest area was high (*r* = –0.62), mangrove forest area tended to be larger at higher latitudes (*r* = 0.38), and population densities tended to be lower at higher latitudes (*r* = –0.31), multicollinearity was low (variance inflation factor = 1.732), thus we considered our LMM analyses to be robust [8, 26].

## Results

### Time Trends in Mangrove Forest Area, Human Population and Fish Abundances

**Fig 2:**
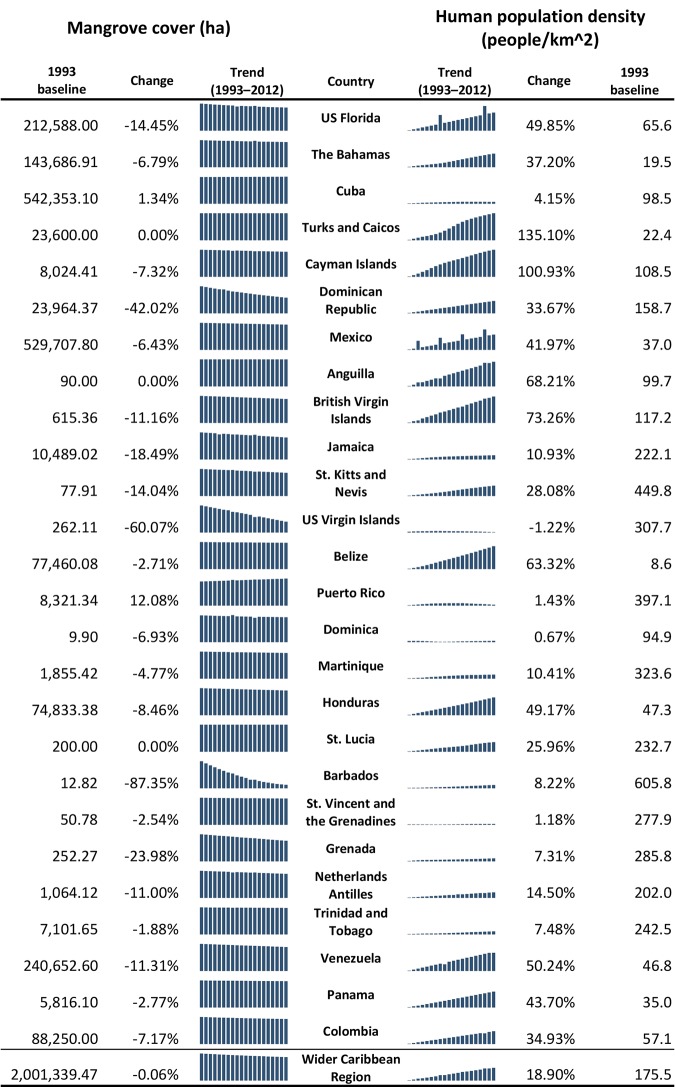
Summary of mangrove extent and human population density data from 1993 to 2012. See Methods for details on where mangrove data and population data are derived. Percent change is the difference between 1993 and 2012.

**Fig 3:**
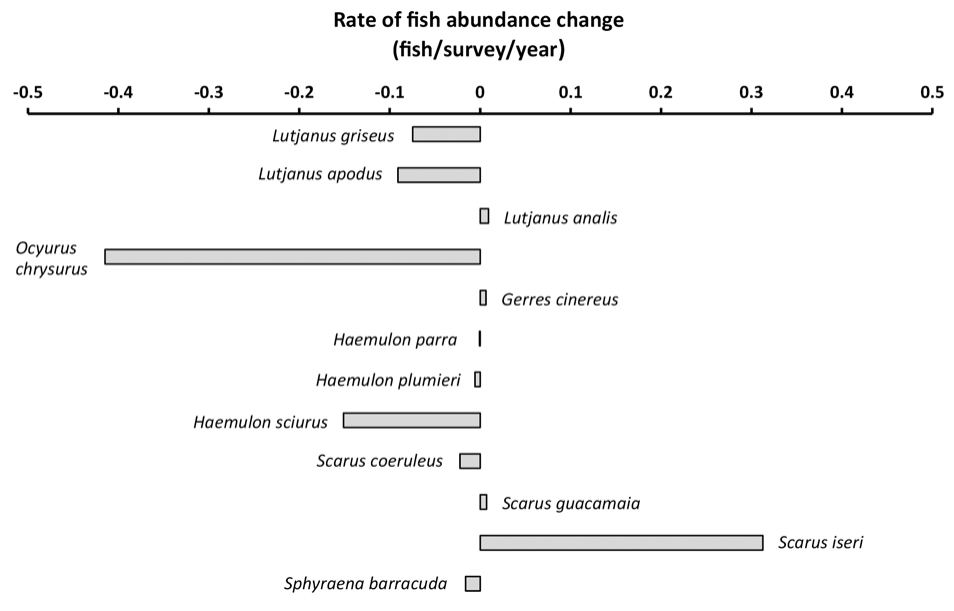
Mean annual rates of change in the abundances on reefs of 12 mangrove-dependent Caribbean fish species. Shown are the coefficients emerging from simple linear regression of the focal species’ abundances versus year. Negative coefficients indicate decline, positive coefficients indicate increase over the period of record (1993–2012). Note that relationships between abundance and time were universally weak (*R* ^2^ values <0.02).

An overall pattern of decline over the 20-year period of record was evident upon compilation of annual mangrove forest area estimates for each country or coastal state (Fig 2). Greatest percent change from 1993 to 2012 for mangrove forest area occurred in Barbados (–87%), U.S. Virgin Islands (–60%) and the Dominican Republic (–42%). Only in Cuba and Puerto Rico were mangrove forest area increases recorded (i.e., 1% and 12%, respectively). Human population density increase predominated among the territories examined, with only one characterized by minor decrease (–1.2%, U.S. Virgin Islands) versus six displaying >50% change since 1993. Numerous data gaps and low sample sizes made identifying clear fish abundance changes over time on a per country/state basis a challenge. However, upon pooling of Caribbean countries/states, overall trends of decline were evident for eight fishes of the 12 species examined (Fig 3). Note that all relationships between abundance and time were universally weak (*R* ^2^ values all <0.02).

### Fish Abundance Limitation

Of the 12 mangrove-dependent fish species examined, quantile regression results (via AIC, Table 1) suggested that mangrove forest area was the primary abundance-limiting factor for eight species on reefs. For all eight, positive fish abundance–mangrove forest area relationships emerged, including for three species belonging to the families Haemulidae, three for Lutjanidae and two for Scaridae. Human population density emerged as the primary limiting factor for two species: yellowtail snapper (*Ocyurus chrysurus*, family Lutjanidae) and yellowfin mojarra (*Gerres cinereus*, family Gerreidae). While yellowtail snapper abundance maxima tended to decline as human population density increased, the opposite pattern emerged for yellowfin mojarra. The two species for which latitude appeared to be the primary limiting factor were great barracuda (*Sphyraena barracuda*, family Sphyraenidae) and striped parrotfish (*Scarus iseri*, family Scaridae): the former’s maximum abundance tended to increase as latitude increased (i.e., increased from south to north), while the latter’s tended to decline.

### Mangrove Forest Area as a Predictor of Mean Reef Fish Abundance

**Fig 4:**
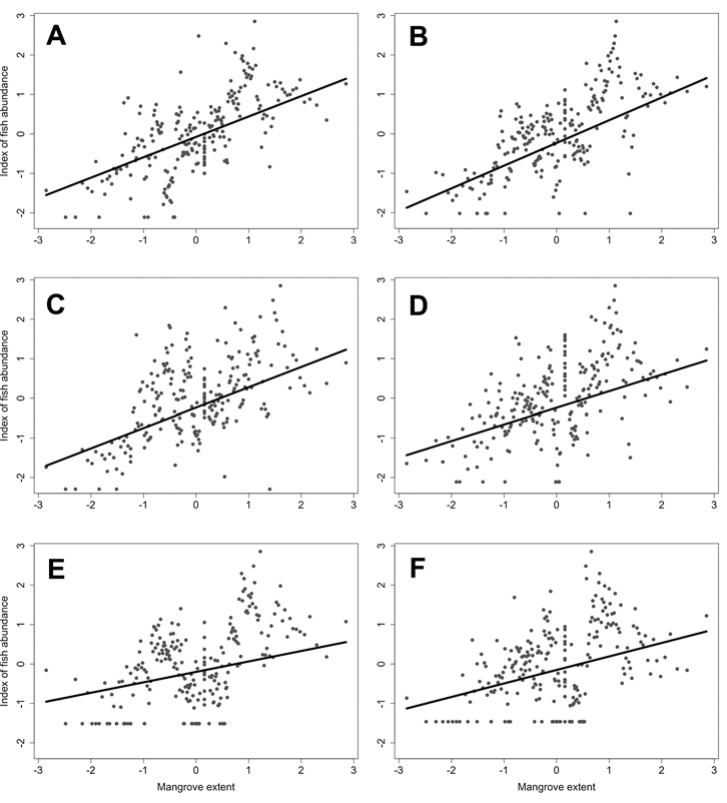
Graphic depiction of relationships between fish abundance and mangrove forest extent (note that mangrove extent on *x*-axis is rank-normal transformed). Shown are species-specific, best-fit linear mixed models determined via Akaike Information Criterion. Top six species are shown based on adjusted *R* ^2^ values. (A) *Haemulon plumierii*, (B) *Haemulon sciurus*, (C) *Sphyraena barracuda*, (D) *Lutjanus apodus*, (E) *Lutjanus griseus*, and (F) *Haemulon parra*. See S2 and S3 Tables for information on models tested.

Linear mixed effects modeling allowed evaluation of the influence of mangrove forest area, in combination with human population density and latitude, as independent factors on mean reef abundance of the 12 focal fish species. Final models emerging from the AIC model selection process (Table 2) indicated that: (1) interaction effects among independent variables were not in the “best approximating” models for any of the species examined (see S3 Table); (2) for 10 species, a positive effect was detected for mangrove area (Fig 4); (3) a human population effect was evident for two species such that the abundance of yellowtail snapper was negatively related to human population density, but yellowfin mojarra showed a positive, relationship; and (4) latitude emerged significant for just one species [yellowtail snapper (*Ocyurus chrysurus*)], whereby fish abundance increased at higher latitudes. The best approximating models had coefficients of determination (adjusted *R* ^2^-values) that ranged from 0.01 to 0.41. The best-fit model for yellowtail snapper had a minor latitude effect, and two other species [bluestriped grunt (*Haemulon sciurus*) and great barracuda (*Sphyraena barracuda*)] had candidate models with evidence to suggest a minor latitude effect (Table 2, S3 Table).

**Table 2:** Linear mixed effects model results (coefficients with one standard error) for reef fish abundances relative to mangrove coverage, human population density, and latitude. Shown are final models of best fit as determined by Akaike Information Criterion (AIC). “Mangrove area” is hectares of mangrove cover (rank transformed) and “Human population density” is people per square kilometer (rank transformed). See S2 Table for initial models. Table results presented here are from the model with the highest AIC weight. See “Remarks” for notes about evidence for other competing models. See S3 Table for more information on those models. *R* ^2^ are adjusted *R* ^2^. na = variable was not in best approximating model.

Family/Species	Intercept	Latitude	Mangrove area	Human population density	*R* ^2^	Remarks
**Lutjanidae**						
*Lutjanus griseus*	–0.20 (0.14)	na	0.27 (0.12)	na	0.16	
*Lutjanus apodus*	–0.24 (0.12)	na	0.51 (0.10)	na	0.25	
*Lutjanus analis*	–0.23 (0.15)	na	0.31 (0.13)	na	0.09	
*Ocyurus chrysurus*	–1.31 (0.38)	0.07 (0.02)	na	–0.43 (0.10)	0.31	Other model possible: population only.
**Gerreidae**						
*Gerres cinereus*	–0.25 (0.16)	na	na	0.08 (0.13)	0.07	Other model possible: mangrove only.
**Haemulidae**						
*Haemulon parra*	–0.15 (0.13)	na	0.34 (0.11)	na	0.14	
*Haemulon sciurus*	–0.23 (0.30)	na	0.58 (0.10)	na	0.40	Other model possible: mangrove + latitude.
*Haemulon plumierii*	–0.08 (0.12)	na	0.52 (0.10)	na	0.41	
**Scaridae**						
*Scarus iseri*	0.03 (0.14)	na	0.03 (0.12)	na	0.01	Other model possible: population only.
*Scarus guacamaia*	–0.10 (0.11)	na	0.28 (0.10)	na	0.11	
*Scarus coeruleus*	–0.12 (0.15)	na	0.13 (0.12)	na	0.07	Other model possible: population only.
**Sphyraenidae**						
*Sphyraena barracuda*	–0.24 (0.14)	na	0.42 (0.12)	na	0.22	Other model possible: mangrove + latitude.

## Discussion

Our results are the first to extend to the entire Caribbean region that which has been observed to operate at smaller-scales—i.e., greater quantities of mangrove habitat generally function to increase the densities of those reef fish species that use these shallow, vegetated habitats as nurseries. Using quantile regression, we revealed that Caribbean mangroves are likely a limiting resource for most (i.e., 8 of 12) of the fishes that Nagelkerken et al. [2] characterized as mangrove-dependent based on their island-scale study. These fishes included several that Dorenbosch et al. [27], Mumby et al. [5], and Nagelkerken et al. [28] found to be in higher average abundance on selected Caribbean reefs located adjacent to mangrove habitat as compared to reefs lacking (or distant from) neighboring mangroves (Table 3).

**Table 3:** Reef fish abundance in relation to mangrove extent. Comparison of present study findings (and analysis methodology) with those of previous studies investigating reef fish abundance in relation to mangrove forests in the Wider Caribbean Region. ANOVA = analysis of variance, LM = linear model, GLM = generalized linear model, LMM = linear mixed model, QR = quantile regression. + = positive relationship with mangroves,– = negative relationship with mangroves, 0 = no relationship with mangroves, letter x indicates fish species was not considered in the analysis, question mark (?) indicates fish species was included in study but there were not enough data to analyze.

	Nagelkerken et al. 2002		Halpern 2004	Mumby et al. 2004	Dorenbosch et al. 2004	Dorenbosch et al. 2007	Present study	
Location	Netherlands Antilles	Puerto Rico/Saba	US and British Virgin Islands	Belize, Mexico	Curaçao	Aruba	Wider Caribbean Region	
Approach	ANOVA	ANOVA	LM	ANOVA	GLM	LMM	QR	LMM
Family/Species								
**Lutjanidae**								
*Lutjanus griseus*	+/0	?	x	x	0	?	+	+
*Lutjanus apodus*	+	+/0	0	+	+	+	+	+
*Lutjanus analis*	+/0	?	x	x	+	x	+	+/0
*Ocyurus chrysurus*	+	+	x	+	+	+	0	0
**Gerreidae**								
*Gerres cinereus*	+/0	–	+	x	–	?	0	0
**Haemulidae**								
*Haemulon parra*	?	?	x	x	?	?	+	+
*Haemulon sciurus*	+	+	x	+	+	0/–	+	+
*Haemulon plumieri*	?	+	x	+	?	0/–	+	+
**Scaridae**								
*Scarus iseri*	+	+	x	+	+/0	+	0	+/0
*Scarus guacamaia*	+/0	?	x	+	?	0/–	+	+/0
*Scarus coeruleus*	+/0	?	x	x	+	x	+	+/0
**Sphyraenidae**								
*Sphyraena barracuda*	+/0	?	x	x	+/0	+	0	+

It is important to note, however, that our resource limitation results are not directly comparable to any previous mangrove-fish studies, largely because others examined only mean (i.e., not maximum) fish densities, which Thomson et al. [29] argued was inappropriate when testing for a resource limitation response. Therefore, our findings might have been expected to differ from those of Halpern [4], the only other relevant study to examine fish abundances along a gradient in mangrove extent and with the stated objective of addressing the resource limitation question. Working in the U.S. and British Virgin Islands and focusing on just two species, Halpern [4] found no relationship between size of nearby mangrove stands and schoolmaster snapper (*Lutjanus apodus*) densities on reefs, but found that yellowfin mojarra (*Gerres cinereus*) densities were positively related to mangrove area. In contrast, our Caribbean-wide results suggest that schoolmaster snapper abundance is indeed limited by reduced mangrove forest area, but that yellowfin mojarra abundance is not (Table 1). The apparent disparity between studies undoubtedly reflects differences in scale, but also in the analytical methods applied. Specifically, Halpern [4], by relying on conventional linear regression in his analyses, did not technically test for a constraining effect and also did not explicitly explore human population density as an alternative, or contributing limiting factor.

At the Caribbean regional scale, mangrove forest area most clearly limited, and was positively correlated with, the abundances of all three *Haemulon* grunt species examined, i.e., white grunt (*H*. *plumieri*), bluestriped grunt (*H*. *sciurus*), and sailors choice (*H*. *parra*). This result is generally consistent with Nagelkerken’s [2] dependency categories for these fishes, and with the findings of Mumby et al. [5] and Nagelkerken et al. [28]. The latter two studies compared “mangrove rich” and “mangrove scarce” reefs and, depending on reef type, observed average white grunt and bluestriped grunt biomass differences at the mangrove-rich sites to exceed mangrove-scarce reefs by 470% and 2600%, respectively (off Belize/Mexico) and by 378 and 203%, respectively (off Grand Cayman). In both cases, differences between neighboring mangrove extent in the mangrove-scarce and mangrove-rich reef sites were dramatic (e.g., off Belize/Mexico, the extent of adjacent mangroves was 46-fold higher for mangrove-rich versus mangrove-scarce reef sites) hence the large differences in fish abundances. Present study results suggest these species’ abundances accrue incrementally as mangrove forest area increases.

Mangrove forest area also appeared to be an abundance-limiting factor (based on the quantile regression) for two of three focal *Scarus* species, namely, rainbow parrotfish (*S*. *guacamaia*), and blue parrotfish (*S*. *coeruleus*), whereas latitude appeared to limit striped parrotfish (*Scarus iseri*) abundance on reefs. More easily comparable with previous studies, however, was our finding that mean abundances (based on the linear mixed modeling) of all three parrotfishes were positively correlated with mangrove forest area, albeit weakly in the case of blue parrotfish and striped parrotfish. Mumby et al. [5] found mean rainbow parrotfish biomass to be significantly higher at the mangrove-rich reef sites they monitored, and Dorenbosch et al. [30] found that juveniles of this species use mangroves exclusively. Working off Curaçao, Dorenbosch et al. [3] found blue parrotfish densities to be highest on reefs adjacent to lagoons harboring seagrass and mangrove habitats (versus reefs far from such lagoons).

Our analyses are the first to suggest that human population density constrains yellowtail snapper (*Ocyurus chrysurus)* abundance, but not the first to suggest that human population density at the regional scale exerts a negative influence on this species. Stallings [8], also using the REEF database, but performing conventional linear correlation analysis on fish presence, found a negative Caribbean-wide human population effect for several predatory fishes, including five considered in the present study: yellowtail snapper; mutton snapper (*Lutjanus analis*); gray snapper (*L*. *griseus*); schoolmaster (*L*. *apodus*) and great barracuda (*Sphyraena barracuda*). While Stallings [8] detected a negative human population effect on the mean presence of all five fishes, we found mangrove forest area to be the primary limiting factor for all three members of genus *Lutjanus* and that latitude was primary limiting factor of great barracuda abundances. Moreover, we also revealed positive relationships between mean fish abundance on reefs and mangrove forest area for all three *Lutjanus* snappers as well as great barracuda. Consistent with the Stallings [8] study, we found that the mean abundance of yellowtail snapper, as was the case with its maxima, was primarily influenced by human population density and to a lesser extent latitude, suggesting that if mangrove extent exerts an influence on this species, it is obscured by other factors such as use of alternative nurseries like seagrass beds [31] and/or anthropogenic factors like fishing pressure. Again, our *Lutjanus* and great barracuda results should not be construed as contradicting those of Stallings [8] because: (1) his focus was on fish presence; (2) he did not consider mangrove forest area *per se* in his analyses; and (3) mangrove extent and human population density often co-occur. Rather, it suggests that, at least for these species, further Caribbean-wide losses of mangrove forest area will have negative consequences on their abundances on reefs, thus protection of mangrove habitat is deserving of consideration given that further human population growth, and all the changes associated with it, seems inevitable.

One of the most challenging issues faced by researchers trying to establish habitat importance to fishes is the difficulty disentangling mangrove effects from those of other co-occurring habitats, especially seagrass beds [32]. Historically, tentative statements and casual observations have been made addressing the distinct roles that mangroves play for fishes, including the mangrove-water interface (nursery, protection for juveniles) [33] and the mangrove trophic contribution (i.e., litterfall detrital- and/or attached algae-based food webs [34]). For their part, seagrass beds often grow in close proximity to mangrove forests and coral reefs, and the habitats are linked by movements of carbon and other materials [35–36]. Because both mangroves and seagrasses serve as nursery areas for juveniles of many species of coral reef fish, several shift between these habitats in complex ways [5, 37]. Mangrove-seagrass interactions, and the natural setting of these ecosystems, complicate not only the assessment of their extent, but also inter-study comparisons. Possessing above-water canopies, mangroves are readily identifiable via remote sensing, and thus country-specific estimates of mangrove forest area are attainable (e.g., Hamilton [38]). But for seagrasses, the majority of which are subtidal, remote sensing accuracy is limited by various factors, including ground-truthing limitations, water depth, water clarity, and more [39]. *The World Atlas of Seagrasses* [39] reports seagrass estimates only in marine protected areas (MPA) for each country in the Caribbean region. Because the number and size of MPAs varies across countries, we were unable to account for seagrass habitat in our country-specific, Caribbean-wide analysis. The only seagrass coverage estimates available are from local-scale studies in which seagrasses have been mapped [39]. To properly account for seagrass effects at the Caribbean regional scale, considerable further work is required to obtain accurate estimates of Caribbean seagrass distribution and abundance. We are aware that the lack of WCR seagrass estimates is a limitation of our study, and at this point it is unknown how incorporating seagrass estimates into our analysis would affect our results.

The present study has several strengths and weaknesses. Strengths include its high number of fish observations, its Caribbean-wide spatial extent, use of data collected over two decades, and the consideration of fish abundances along a mangrove forest area gradient, as opposed to “binary” contrasts between reef systems with versus without adjacent vegetated habitats. Present study weaknesses include the lack of quantitative data on fish sizes, mangrove-to-reef distance measurements, estimates of seagrass and coral reef areas, and quantification of fishing effort per se. Yet, despite these limitations, consistent patterns of reef fish abundance limitation by, and/or positive correlation with, mangrove forest area emerged for most of the species examined. Therefore, our results contrast with the conclusions drawn by Saenger et al. [7], who conducted a literature review of mangrove and seagrass linkages to fisheries production for the United Nations Food and Agriculture Organization. After considering more than 200 published studies, including most of those considered here, Saenger et al. [7] were unconvinced of a clear fish-mangrove linkage, either within or among regions, largely because previous study findings appeared mixed and their spatiotemporal scale was limited. However, our Caribbean regional results contradict the Saenger et al. [7] conclusion that “It is misleading to generalize that there is a prevailing effect of mangrove and seagrass areas as nursery areas or enhancers of survival at other life stages of most of the species that occur at some time within them”. Not only do our results suggest otherwise, but from a precautionary approach standpoint, it seems prudent to assume a positive reef fish–mangrove linkage exists for those species that occupy mangroves as juveniles, at least until conclusive evidence to the contrary is gathered. We suggest that recent work questioning the mangrove-reef fish subsidy effect (e.g., Saenger et al. [7]) likely reflects a failure to: (1) focus analyses on species that use mangroves as nurseries; (2) consider more than the mean fish abundance response to mangrove forest area; and/or (3) to quantitatively account for potentially confounding human impacts, such as fishing pressure.

Our study is another of several that underscore the merit of volunteer, citizen-scientist organizations to provide large, valuable data sets that extend well beyond the spatiotemporal scope of most conventional governmental or academic research entities [8, 40–43]. In light of increasing regional human population growth, climate change, ocean acidification, and sea level rise, the REEF fish data are valuable for addressing many challenging questions surrounding Caribbean marine fish resources.

In summary, our two-stage approach afforded the opportunity to examine questions surrounding mangroves as fish habitat from two angles. Results from the quantile regression suggested that mangrove area is potentially a limiting factor to abundance for 8 of the 12 reef fish examined here. Furthermore, we found that mangrove forest area alone may be the best predictor for abundances of at least six of the reef fish we examined, even after accounting for possible human population pressures; mangrove cover combined with other factors emerged for 11 of the 12 species. Our results suggest that if negative trends of mangrove distribution continue into the future, driven in part by large-scale conversion of mangrove forest for aquaculture, agriculture, infrastructure, and tourism, and by other processes including pollution and natural disasters [18, 19, 35], several Caribbean fish populations can also be expected to concurrently decline, even in the absence of other depleting forces such as fishing. Mangrove forest area and human population density have clearly been moving in opposite directions in the Caribbean region over the last two decades, and it seems likely that most of the species examined here will follow suite.

## Supporting Information

S1 Table Summary of REEF citizen-science fish surveys per country per year, included in each fish abundance analysis. (DOCX)

S2 Table Initial set of candidate models for examining factors linking fish abundance with mangrove extent, human population density, latitude, and their interactions. (DOCX)

S3 Table Results of Akaike information criterion (AIC) approach and summary outputs of any competing models. (DOCX)

## References

[1] Parrish JD (1989) Fish communities of interacting shallow water habitats in tropical oceanic regions. Mar Ecol Prog Ser. 58:143–160. Available: 10.3354/meps058143.

[2] Nagelkerken I, Roberts CM, van der Velde G, Dorenbosch M, van Riel MC, Cocheret de la Morinière E, et al (2002) How important are mangroves and seagrass beds for coral-reef fish? The nursery hypothesis tested on an island scale. Mar Ecol Prog Ser. 244:299–305. Available: 10.3354/meps244299.

[3] Dorenbosch M, van Riel MC, Nagelkerken I, van der Velde G (2004) The relationship of reef fish to the proximity of mangrove and seagrass nurseries. Estuar Coast Shelf Sci. 60:37–48. Available: 10.1016/j.ecss.2003.11.018.

[4] Halpern B (2004) Are mangroves a limiting resource for two coral reef fishes? Mar Ecol Prog Ser. 272:93–98. Available: 10.3354/meps272093.

[5] Mumby PJ, Edwards AJ, Arias-Gonzalez JE, Lindeman KC, Blackwell PG, Gall A, et al (2004) Mangroves enhance the biomass of coral reef fish communities in the Caribbean. Nature. 427:533–536. PubMed. Available: 10.1038/nature02286. 14765193

[6] Jones DL, Walter JF, Brooks EN, Serafy JE (2010) Connectivity through ontogeny: fish population linkages among mangrove and coral reef habitats. Mar Ecol Prog Ser. 401:245–258. Available: 10.3354/meps08404.

[7] Saenger P, Gartside D, Funge-Smith S (2013) A review of mangrove and seagrass ecosystems and their linkage to fisheries and fisheries management. FAO Regional Office for Asia and the Pacific, Bangkok, Thailand, RAP Publication 2013/09. 74 p.

[8] Stallings CD (2009) Fishery-independent data reveal negative effect of human population density on Caribbean predatory fish communities. PLoS ONE. 4(5):e5333 PubMed. Available: 10.1371/journal.pone.0005333 19421312 PMC2672166

[9] Halpern BS, Longo C, Hardy D, McLeod KL, Samhouri JF, Katona SK, et al (2012) An index to assess the health and benefits of the global ocean. Nature. 488:615–620. PubMed. 10.1038/nature11397 22895186

[10] Terrell JW, Cade BS, Carpenter J, Thompson JM (1996) Modeling stream fish habitat limitations from wedged-shaped patterns of variation in standing stock. Trans Am Fish Soc. 125:104–117. Available: 10.1577/1548-8659(1996)125<0104:MSFHLF>2.3.CO;2.

[11] Cade BS, Terrell JW, Schroeder RL (1999) Estimating effects of limiting factors with regression quantiles. Ecology. 80:311–323. Available: 10.1890/0012-9658(1999)080[0311:EEOLFW]2.0.CO;2.

[12] Cade BS, Noon BR (2003) A gentle introduction to quantile regression for ecologists. Front Ecol Environ. 1:412–420. Available: 10.1890/1540-9295(2003)001[0412:AGITQR]2.0.CO;2.

[13] Austin M (2007) Species distribution models and ecological theory: A critical assessment and some possible new approaches. Ecol Modell. 200:1–19. Available: 10.1016/j.ecolmodel.2006.07.005.

[14] Planque B, Buffaz L (2008) Quantile regression models for fish recruitment–environment relationships: four case studies. Mar Ecol Prog Ser. 357:213–223. Available: 10.3354/meps07274.

[15] Schmitt EF, Sullivan KM (1996) Analysis of a volunteer method for collecting fish presence and abundance data in the Florida Keys. Bull Mar Sci. 59(2):404–416.

[16] Pattengill-Semmens CV, Semmens BX (2003) Conservation and management applications of the REEF volunteer fish monitoring program. Environmental Monitoring and Assessment Journal. 81:43–50. PubMed. Available: 10.1023/A:1021300302208. 12620003

[17] Wolf JR, Pattengill-Semmens CV (2013) Estimating fish populations from order-of-magnitude surveys. CCOFI Rep. 54:127–140.

[18] FAO (2007a) Mangroves of North and Central America 1980–2005: Country Reports. Forest Resources Assessment Programme Working Paper 138. FAO, Rome. 34 p. Available: ftp://ftp.fao.org/docrep/fao/010/ai446t/ai446t00.pdf.

[19] FAO (2007b) Mangroves of South America 1980–2005: Country Reports. Forest Resources Assessment Programme Working Paper 140. FAO, Rome. 46 p. Available: ftp://ftp.fao.org/docrep/fao/010/ai448t/ai448t00.pdf.

[20] Burnham KP, Anderson DR (2002) Model selection and multimodel inference: a practical information-theoretic approach Second edition Springer, New York, NY.

[21] Koenker R (2013) quantreg: Quantile Regression. R package version 5.05. Available: http://CRAN.R-project.org/package=quantreg.

[22] Pinheiro J, Bates D, DebRoy S, Sarkar D, and the R Development Core Team (2013) nlme: linear and nonlinear mixed effects models. R package version 3.1–108.

[23] Krueger C, Tian L (2004) A comparison of general linear mixed model and repeated meastures ANOVA using a dataset with multiple missing data points. Biological Research for Nursing. 6(2):151–157. Available: 10.1177/1099800404267682488 p. 15388912

[24] Faraway JJ (2006) Extending the linear model with R Generalized linear, mixed effects and nonparametric regression models. Chapman & Hall/CRC Boca Raton, FL. 301 p.

[25] Conover WJ (2012) The rank transformation—an easy and intuitive way to connect many nonparametric methods to their parametric counterparts for seamless teaching introductory statistics courses. Wiley Interdiscip Rev Comput Stat. 4:432–438. Available: 10.1002/wics.1216.

[26] O’Brien RM (2007) A caution regarding rules of thumb for variance inflation factors. Qual Quant. 41:673–690. 10.1007/s11135-006-9018-6.

[27] Dorenbosch M, Verberk WCEP, Nagelkerken I, van der Velde G (2007) Influence of habitat configuration on connectivity between seagrass beds, mangroves and coral reefs. Mar Ecol Prog Ser. 334:103–116. Available: 10.3354/meps334103.

[28] Nagelkerken I, Grol MGG, Mumby PJ (2012) Effects of marine reserves versus nursery habitat availability on structure of reef fish communities. PLoS ONE. 7:e36906 10.1371/journal.pone.0036906 22675474 PMC3366965

[29] Thomson JD, Weiblen G, Thomson BA, Alfaro S, Legendre P (1996) Untangling multiple factors in spatial distributions: Lilies, gophers, and rocks. Ecology. 77:1698–1715. Available: 10.2307/2265776.

[30] Dorenbosch M, Grol MGG, Nagelkerken I, van der Velde G (2006) Seagrass beds and mangroves as potential nurseries for the threatened Indo-Pacific humphead wrasse, Cheilinus undulatus and Caribbean rainbow parrotfish, Scarus guacamaia. Biological Conservation, 129:277–282.

[31] Verweij MC, Nagelkerken I, Hans I, Ruseler SM, Mason PRD (2008) Seagrass nurseries contribute to coral reef fish populations. Limnology and Oceanography. 53:1540–1547.

[32] Nagelkerken I, Sheaves M, Baker R, Connolly RM (2015) The seascape nursery: a novel spatial approach to identify and manage nurseries for coastal marine fauna. Fish and Fisheries. 16:362–371.

[33] Nagelkerken I (2009) Evaluation of nursery function of mangroves and seagrass beds for tropical decapods and reef fishes: patterns and underlying mechanisms In: Nagelkerken I (ed.) Ecological connectivity among tropical coastal ecosystems. Springer Science and Business Media, Dordrecht, the Netherlands p. 357–399.

[34] Hyndes GA, Nagelkerken I, McLeod RJ, Connolly RM, Lavery PS, Vanderklift MA (2014) Mechanisms and ecological role of carbon transfer within coastal seascapes. Biological Reviews. 89:232–254. 10.1111/brv.12055 23980752

[35] Lin GH, Banks T, Sternberg LDL (1991) Variation in δ13C values for the seagrass Thalassia testudinum and its relation to mangrove carbon. Aquat Bot. 40:333–341. Available: 10.1016/0304-3770(91)90079-K.

[36] Hemminga MA, Slim FJ, Kazungu J, Ganssen GM, Nieuwenhuize J, Kruyt NM (1994) Carbon outwelling from a mangrove forest with adjacent seagrass beds and coral reefs (Ganzi Bay, Kenya). Mar Ecol Prog Ser. 106:291–301. Available: 10.3354/meps106291.

[37] Grol MGG, Rypel AL, Nagelkerken I (2014) Growth potential and predation risk drive ontogenetic shifts among nursery habitats in a coral reef fish. Marine Ecology Progress Series. 502:229–244.

[38] Hamilton S (2013) Assessing the role of commercial aquaculture in displacing mangrove forest. Bull Mar Sci. 89:585–601. Available: 10.5343/bms.2012.1069.

[39] Green EP, Short FT (2003) The world atlas of seagrasses University of California Press Berkley, California. 298 p.

[40] Bell CD, Blumenthal JM, Austin TJ, Ebanks-Petrie G, Broderick AC, Godley BJ (2008) Harnessing recreational divers for the collection of sea turtle data around the Cayman Islands. Tourism in Marine Environments. 5:245–257. Available: 10.3727/154427308788714768.

[41] Goffredo S, Pensa F, Neri P, Orlandi A, Gagliardi MS, Velardi A, et al (2010) Unite research with what citizens do for fun: “recreational monitoring” of marine biodiversity. Ecol Appl. 20:2170–2187. PubMed. Available: 10.1890/09-1546.1. 21265450

[42] Ward-Paige CA, Lotze HK. 2011 Assessing the value of recreational divers for censusing elasmobranchs. PLoS ONE. 6:e25609 PubMed. Available: 10.1371/journal.pone.0025609 22016771 PMC3189927

[43] Vianna GMS, Meekan MG, Bornovski TH, Meeuwig JJ (2014) Acoustic telemetry validates a citizen science approach for monitoring sharks on coral reefs. PLoS ONE. 9(4):e95565 PubMed. Available: 10.1371/journal.pone.0095565 24760081 PMC3997401

