# Supplementary material for: Mangroves Enhance Reef Fish Abundance at the Caribbean Regional Scale
Source: PLoS One. 2015 Nov 4;10(11):e0142022. doi: 10.1371/journal.pone.0142022 (PMC4633132; doi:10.1371/journal.pone.0142022)
Supplement: S1 Table — (DOCX) [file pone.0142022.s001.docx]

**S1 Table. Summary of REEF citizen-science fish surveys (*n*) per country per year, included in each fish abundance analysis. For years where n < 10, data were omitted.**

| Country | 1993 | 1994 | 1995 | 1996 | 1997 | 1998 | 1999 | 2000 | 2001 | 2002 | 2003 | 2004 | 2005 | 2006 | 2007 | 2008 | 2009 | 2010 | 2011 | 2012 |
| --- | --- | --- | --- | --- | --- | --- | --- | --- | --- | --- | --- | --- | --- | --- | --- | --- | --- | --- | --- | --- |
| Anguilla | 0 | 0 | 0 | 0 | 0 | 0 | 0 | 0 | 0 | 0 | 0 | 1 | 0 | 0 | 66 | 19 | 19 | 0 | 0 | 0 |
| Barbados | 0 | 0 | 0 | 0 | 0 | 0 | 0 | 0 | 0 | 0 | 134 | 0 | 0 | 0 | 0 | 0 | 0 | 0 | 0 | 4 |
| Belize | 0 | 33 | 17 | 40 | 0 | 0 | 0 | 6 | 69 | 1 | 136 | 2 | 0 | 193 | 33 | 47 | 73 | 67 | 43 | 4 |
| British Virgin Islands | 0 | 1 | 45 | 56 | 54 | 51 | 9 | 18 | 1 | 49 | 116 | 126 | 42 | 37 | 84 | 56 | 25 | 37 | 59 | 103 |
| Cayman Islands | 0 | 35 | 1 | 13 | 89 | 61 | 187 | 128 | 144 | 216 | 131 | 178 | 261 | 221 | 128 | 105 | 93 | 93 | 172 | 132 |
| Colombia | 0 | 0 | 0 | 0 | 0 | 0 | 0 | 0 | 0 | 163 | 1 | 23 | 0 | 0 | 0 | 23 | 8 | 0 | 16 | 64 |
| Cuba | 0 | 0 | 0 | 3 | 0 | 0 | 0 | 0 | 64 | 0 | 0 | 0 | 0 | 0 | 0 | 0 | 0 | 0 | 0 | 2 |
| Dominica | 0 | 0 | 0 | 0 | 0 | 0 | 0 | 49 | 0 | 0 | 0 | 111 | 0 | 0 | 2 | 0 | 32 | 75 | 25 | 10 |
| Dominican Republic | 0 | 52 | 2 | 35 | 12 | 1 | 0 | 0 | 6 | 6 | 0 | 0 | 30 | 0 | 4 | 0 | 0 | 0 | 0 | 0 |
| Grenada | 0 | 0 | 0 | 0 | 0 | 0 | 0 | 0 | 0 | 100 | 11 | 0 | 0 | 1 | 0 | 0 | 82 | 1 | 0 | 5 |
| Honduras | 0 | 0 | 0 | 0 | 0 | 0 | 48 | 113 | 114 | 236 | 100 | 108 | 74 | 53 | 215 | 68 | 138 | 209 | 189 | 29 |
| Jamaica | 0 | 0 | 0 | 0 | 0 | 0 | 0 | 2 | 0 | 0 | 0 | 4 | 0 | 0 | 0 | 15 | 13 | 0 | 0 | 42 |
| Martinique | 0 | 0 | 0 | 0 | 0 | 0 | 0 | 0 | 0 | 0 | 0 | 0 | 0 | 94 | 0 | 0 | 0 | 0 | 0 | 0 |
| Mexico | 0 | 0 | 0 | 0 | 0 | 20 | 73 | 201 | 133 | 165 | 257 | 308 | 133 | 213 | 261 | 326 | 334 | 347 | 320 | 342 |
| Netherlands Antilles | 0 | 0 | 128 | 43 | 52 | 178 | 316 | 283 | 694 | 481 | 998 | 1215 | 696 | 1143 | 1379 | 990 | 747 | 587 | 658 | 606 |
| Panama | 0 | 0 | 0 | 0 | 0 | 0 | 0 | 0 | 0 | 30 | 0 | 0 | 0 | 0 | 0 | 88 | 0 | 0 | 0 | 88 |
| Puerto Rico | 0 | 0 | 0 | 0 | 0 | 0 | 0 | 0 | 192 | 179 | 48 | 14 | 31 | 15 | 0 | 0 | 0 | 0 | 4 | 0 |
| St. Kitts and Nevis | 0 | 0 | 0 | 0 | 0 | 22 | 22 | 0 | 0 | 16 | 0 | 0 | 6 | 9 | 0 | 0 | 5 | 8 | 0 | 47 |
| St. Lucia | 0 | 0 | 0 | 0 | 0 | 0 | 0 | 0 | 0 | 0 | 12 | 8 | 0 | 2 | 0 | 0 | 23 | 0 | 0 | 0 |
| St. Vincent and the Grenadines | 0 | 0 | 14 | 0 | 0 | 0 | 0 | 18 | 124 | 51 | 203 | 177 | 206 | 97 | 92 | 152 | 156 | 22 | 1 | 0 |
| The Bahamas | 0 | 32 | 17 | 64 | 73 | 65 | 4 | 191 | 181 | 203 | 136 | 331 | 192 | 245 | 686 | 137 | 69 | 50 | 182 | 147 |
| Trinidad and Tobago | 0 | 0 | 0 | 0 | 0 | 0 | 4 | 119 | 18 | 15 | 9 | 16 | 47 | 55 | 31 | 0 | 0 | 25 | 0 | 0 |
| Turks and Caicos | 0 | 0 | 38 | 15 | 26 | 25 | 18 | 89 | 32 | 21 | 136 | 8 | 27 | 26 | 37 | 255 | 29 | 12 | 2 | 0 |
| U.S. Florida | 22 | 254 | 172 | 124 | 403 | 449 | 465 | 568 | 1082 | 1596 | 1852 | 937 | 863 | 1623 | 1220 | 699 | 829 | 863 | 763 | 615 |
| U.S. Virgin Islands | 0 | 0 | 0 | 21 | 0 | 42 | 100 | 70 | 204 | 50 | 118 | 138 | 302 | 61 | 6 | 11 | 91 | 36 | 7 | 60 |
| Venezuela | 0 | 0 | 2 | 0 | 0 | 6 | 125 | 17 | 18 | 132 | 4 | 0 | 0 | 1 | 2 | 21 | 46 | 19 | 18 | 33 |
