# Supplementary material for: Mangroves Enhance Reef Fish Abundance at the Caribbean Regional Scale
Source: PLoS One. 2015 Nov 4;10(11):e0142022. doi: 10.1371/journal.pone.0142022 (PMC4633132; doi:10.1371/journal.pone.0142022)
Supplement: S2 Table — (DOCX) [file pone.0142022.s002.docx]

**S2 Table. Initial set of candidate models for examining factors linking fish abundance with mangrove extent, human population density, latitude, and their interactions. For all 12 species examined, the Akaike Information Criterion approach used began with this set of candidate models. For all models with interactions, the hierarchy principle was honored.**

| Model | Model description |
| --- | --- |
| 1 | Mangrove only |
| 2 | Human density only |
| 3 | Latitude only |
| 4 | Mangrove and human density |
| 5 | Mangrove and latitude |
| 6 | Human density and latitude |
| 7 | Mangrove and human density interaction |
| 8 | Mangrove and latitude interaction |
| 9 | Human density and latitude interaction |
| 10 | Mangrove, human density, and latitude interaction |
