# Supplementary material for: Mangroves Enhance Reef Fish Abundance at the Caribbean Regional Scale
Source: PLoS One. 2015 Nov 4;10(11):e0142022. doi: 10.1371/journal.pone.0142022 (PMC4633132; doi:10.1371/journal.pone.0142022)
Supplement: S3 Table — (DOCX) [file pone.0142022.s003.docx]

**S3 Table. Results of Akaike information criterion (AIC) approach and summary outputs of any competing models (differences from best AIC <2, in bold). Results of “best approximating” models are found in Table 2. Model numbers refer to the models in Online Appendix S2. Difference = the AIC difference between the model and the “best approximating” model; AIC weight = the calculated probability that the model is the “best approximating.”**

***Lutjanus griseus:***

| Model | AIC | Difference | AIC weight |
| --- | --- | --- | --- |
| 1 | 402.16 | 0.00 | 58.48 |
| 3 | 405.04 | 2.88 | 13.89 |
| 2 | 405.45 | 3.29 | 11.31 |
| 4 | 406.17 | 4.00 | 7.90 |
| 5 | 406.88 | 4.71 | 5.54 |
| 6 | 409.16 | 6.99 | 1.77 |
| 7 | 410.65 | 8.48 | 0.84 |
| 8 | 413.38 | 11.22 | 0.21 |
| 9 | 416.16 | 14.00 | 0.05 |
| 10 | 431.34 | 29.18 | 0.00 |

Competing model:

None

***Lutjanus apodus:***

| Model | AIC | Difference | AIC weight |
| --- | --- | --- | --- |
| 1 | 501.45 | 0.00 | 84.20 |
| 4 | 505.62 | 4.17 | 10.46 |
| 5 | 507.62 | 6.17 | 3.85 |
| 7 | 510.30 | 8.85 | 1.01 |
| 2 | 512.78 | 11.33 | 0.29 |
| 8 | 514.85 | 13.40 | 0.10 |
| 3 | 516.16 | 14.71 | 0.05 |
| 6 | 517.24 | 15.79 | 0.03 |
| 9 | 523.84 | 22.39 | 0.00 |
| 10 | 534.67 | 33.22 | 0.00 |

Competing model:

None

***Sphyraena barracuda:***

| Model | AIC | Difference | AIC weight |
| --- | --- | --- | --- |
| 1 | 458.74 | 0.00 | 36.51 |
| **5** | **458.99** | **0.26** | **32.14** |
| 6 | 461.50 | 2.76 | 9.19 |
| 4 | 461.56 | 2.83 | 8.88 |
| 2 | 462.39 | 3.65 | 5.89 |
| 3 | 462.57 | 3.83 | 5.38 |
| 8 | 466.22 | 7.49 | 0.86 |
| 7 | 466.40 | 7.66 | 0.79 |
| 9 | 467.99 | 9.25 | 0.36 |
| 10 | 485.38 | 26.64 | 0.00 |

Competing models:

Model 5 (*R*^2^ = 0.44)

|  | Coefficient | SE |
| --- | --- | --- |
| Intercept | -1.4184011 | 0.4343655 |
| Mangrove | 0.3477838 | 0.1079844 |
| Latitude | 0.0717185 | 0.0251408 |

***Gerres cinereus:***

| Model | AIC | Difference | AIC weight |
| --- | --- | --- | --- |
| 2 | 519.54 | 0.00 | 41.35 |
| **1** | **519.71** | **0.17** | **37.94** |
| 4 | 522.59 | 3.05 | 9.00 |
| 3 | 522.77 | 3.23 | 8.22 |
| 6 | 526.58 | 7.04 | 1.22 |
| 5 | 526.77 | 7.23 | 1.11 |
| 7 | 526.84 | 7.30 | 1.08 |
| 9 | 533.36 | 13.82 | 0.04 |
| 8 | 533.65 | 14.11 | 0.04 |
| 10 | 552.36 | 32.82 | 0.00 |

Competing model:

Model 1 (*R*^2^ = 0.05)

|  | Coefficient | SE |
| --- | --- | --- |
| Intercept | -0.22086707 | 0.1513094 |
| Mangrove | 0.05832585 | 0.1267676 |

***Scarus guacamaia:***

| Model | AIC | Difference | AIC weight |
| --- | --- | --- | --- |
| 1 | 543.24 | 0.00 | 74.73 |
| 4 | 546.20 | 2.96 | 17.03 |
| 2 | 549.18 | 5.94 | 3.84 |
| 5 | 550.90 | 7.66 | 1.63 |
| 7 | 551.07 | 7.82 | 1.50 |
| 3 | 551.67 | 8.43 | 1.10 |
| 6 | 556.08 | 12.84 | 0.12 |
| 8 | 558.12 | 14.87 | 0.04 |
| 9 | 562.78 | 19.54 | 0.00 |
| 10 | 579.22 | 35.98 | 0.00 |

Competing model:

None

***Haemulon parra:***

| Model | AIC | Difference | AIC weight |
| --- | --- | --- | --- |
| 1 | 492.56 | 0.00 | 67.44 |
| 4 | 494.87 | 2.31 | 21.29 |
| 5 | 497.95 | 5.38 | 4.57 |
| 3 | 499.28 | 6.72 | 2.35 |
| 7 | 499.35 | 6.79 | 2.27 |
| 2 | 499.95 | 7.39 | 1.68 |
| 6 | 503.61 | 11.04 | 0.27 |
| 8 | 505.16 | 12.59 | 0.12 |
| 9 | 510.35 | 17.79 | 0.01 |
| 10 | 524.55 | 31.99 | 0.00 |

Competing model:

None

***Haemulon sciurus:***

| Model | AIC | Difference | AIC weight |
| --- | --- | --- | --- |
| 1 | 409.91 | 0.00 | 55.19 |
| **5** | **410.81** | **0.91** | **35.10** |
| 4 | 414.26 | 4.35 | 6.28 |
| 8 | 415.82 | 5.91 | 2.87 |
| 7 | 419.21 | 9.31 | 0.53 |
| 3 | 426.18 | 16.27 | 0.02 |
| 6 | 426.56 | 16.65 | 0.01 |
| 2 | 426.97 | 17.07 | 0.01 |
| 9 | 432.40 | 22.49 | 0.00 |
| 10 | 435.70 | 25.80 | 0.00 |

Competing model:

Model 5 (*R*^2^ = 0.56)

|  | Coefficient | SE |
| --- | --- | --- |
| Intercept | -1.1889867 | 0.3546735 |
| Mangrove | 0.5184231 | 0.0898519 |
| Latitude | 0.0584686 | 0.0204870 |

***Ocyurus chrysurus:***

| Model | AIC | Difference | AIC weight |
| --- | --- | --- | --- |
| 6 | 547.33 | 0.00 | 48.18 |
| **2** | **547.64** | **0.31** | **41.34** |
| 4 | 551.10 | 3.76 | 7.33 |
| 9 | 554.50 | 7.16 | 1.34 |
| 7 | 555.69 | 8.36 | 0.74 |
| 1 | 556.32 | 8.99 | 0.54 |
| 5 | 557.43 | 10.09 | 0.31 |
| 3 | 558.18 | 10.85 | 0.21 |
| 8 | 564.05 | 16.71 | 0.01 |
| 10 | 576.35 | 29.01 | 0.00 |

Competing model:

Model 2 (*R*^2^ = 0.16)

|  | Coefficient | SE |
| --- | --- | --- |
| Intercept | -0.1984042 | 0.1311509 |
| Population | -0.4939052 | 0.1129449 |

***Scarus iseri:***

| Model | AIC | Difference | AIC weight |
| --- | --- | --- | --- |
| 1 | 633.70 | 0.00 | 39.45 |
| **2** | **633.75** | **0.06** | **38.38** |
| 3 | 636.76 | 3.07 | 8.50 |
| 4 | 637.59 | 3.89 | 5.63 |
| 9 | 638.59 | 4.90 | 3.41 |
| 7 | 639.23 | 5.53 | 2.48 |
| 5 | 640.91 | 7.21 | 1.07 |
| 6 | 641.00 | 7.30 | 1.02 |
| 8 | 647.05 | 13.35 | 0.05 |
| 10 | 658.60 | 24.91 | 0.00 |

Competing model:

Model 2 (*R*^2^ = 0.01)

|  | Coefficient | SE |
| --- | --- | --- |
| Intercept | 0.03019992 | 0.1430077 |
| Population | -0.01240220 | 0.1254379 |

***Haemulon plumierii:***

| Model | AIC | Difference | AIC weight |
| --- | --- | --- | --- |
| 1 | 392.09 | 0.00 | 73.59 |
| 5 | 395.46 | 3.37 | 13.62 |
| 4 | 395.96 | 3.87 | 10.61 |
| 7 | 400.10 | 8.02 | 1.34 |
| 8 | 401.40 | 9.31 | 0.70 |
| 3 | 405.51 | 13.42 | 0.09 |
| 2 | 407.38 | 15.30 | 0.04 |
| 6 | 408.86 | 16.77 | 0.02 |
| 9 | 410.97 | 18.89 | 0.01 |
| 10 | 422.51 | 30.42 | 0.00 |

Competing model:

None

***Lutjanus analis:***

| Model | AIC | Difference | AIC weight |
| --- | --- | --- | --- |
| 1 | 518.02 | 0.00 | 68.40 |
| 2 | 521.26 | 3.24 | 13.50 |
| 4 | 521.85 | 3.84 | 10.04 |
| 3 | 524.08 | 6.06 | 3.30 |
| 5 | 524.56 | 6.55 | 2.59 |
| 7 | 525.94 | 7.93 | 1.30 |
| 6 | 527.10 | 9.09 | 0.73 |
| 8 | 530.92 | 12.91 | 0.11 |
| 9 | 533.45 | 15.43 | 0.03 |
| 10 | 550.65 | 32.63 | 0.00 |

Competing model:

None

***Scarus coeruleus:***

| Model | AIC | Difference | AIC weight |
| --- | --- | --- | --- |
| 1 | 408.83 | 0.00 | 48.22 |
| **2** | **409.82** | **0.99** | **29.38** |
| 3 | 411.77 | 2.94 | 11.09 |
| 4 | 412.93 | 4.09 | 6.23 |
| 7 | 415.22 | 6.39 | 1.98 |
| 5 | 415.50 | 6.67 | 1.72 |
| 6 | 416.16 | 7.33 | 1.24 |
| 9 | 421.27 | 12.44 | 0.10 |
| 8 | 422.57 | 13.74 | 0.05 |
| 10 | 441.07 | 32.23 | 0.00 |

Competing model:

Model 2 (*R*^2^ = 0.02)

|  | Coefficient | SE |
| --- | --- | --- |
| Intercept | -0.13235310 | 0.1488546 |
| Population | -0.04497999 | 0.1161276 |
